# Melatonin Alleviates Acute Sleep Deprivation-Induced Memory Loss in Mice by Suppressing Hippocampal Ferroptosis

**DOI:** 10.3389/fphar.2021.708645

**Published:** 2021-07-16

**Authors:** Xintong Wang, Zixu Wang, Jing Cao, Yulan Dong, Yaoxing Chen

**Affiliations:** Neurobiology Laboratory, College of Veterinary Medicine, China Agricultural University, Beijing, China

**Keywords:** sleep deprivation, ferroptosis, melatonin, hippocampus, memory loss, intracellular signaling pathway, MT2

## Abstract

**Objectives:** Memory decline caused by insufficient sleep is a critical public health issues and currently lacks effective treatments. This study objective was to explore alleviative effect of melatonin on sleep deprivation (SD)-induced deficiencies in learning and memory.

**Materials and Methods:** A continuous 72 h SD mouse model, with or without melatonin or Fer-1 supplementation were established. The changes of cognitive function, iron homeostasis, lipid peroxidation and intracellular signal pathways in mice were detected by Morris water maze, antioxidant assay, immunohistochemistry, western blot, RT-PCR and Prussian blue staining. *In vitro*, we treated HT-22 cells with ferroptosis inducer (Erastin) to further explore the specific mechanism of melatonin in ferroptosis.

**Results:** Mice subjected to SD had significantly elevated latency and path length to reach hidden platform, as well as a decrease in number of entries and time spent in the target zone when the hidden platform was removed (*p* < 0.05). Nevertheless, supplementation with ferroptosis inhibitor (Fer-1) mitigated the memory impairment associated with SD. Further evaluation revealed an up-regulation of intracellular iron accumulation, transferrin receptor 1 and divalent metal transporter 1 expression and ROS and MDA production, and a down-regulation of ferroportin and antioxidant enzyme (GPX4 and SOD) expression in SD mice. SD decreased expression of MT2 receptor rather than of MT1, and inhibited ERK/Nrf2 signaling activation in the hippocampus (*p* < 0.05). In contrast, the aforementioned SD-inductions were reversed by supplementation using 20 and 40 mg/kg melatonin in SD mice. *In vitro*, melatonin pretreatment reversed Erastin-induced ferroptosis, abnormalities in iron transporter protein and antioxidant enzyme expression and suppression of ERK/Nrf2 signaling in HT-22 cells, however this protective effect of melatonin was blocked by MT2-, ERK- and Nrf2-specific antagonists (*p* < 0.05).

**Conclusion:** Our finding suggested SD may induce ferroptosis, in turn leading to cognitive deficits. Melatonin alleviated memory loss and hippocampal ferroptosis caused by acute SD through binding to the MT2 receptor to activate ERK/Nrf2 signaling.

## Introduction

In 2021, the journal of Science released a new edition of “125 scientific questions at the forefront of the world”. One of the questions is why do we need sleep, which undoubtedly illustrates the importance of sleep for human health. With the surge of social pressure, more people choose to sacrifice sleep time to study, work and so on. This causes a series of health crises, such as cognitive impairment (Leng et al., 2017) and cardiovascular damage (Shahrbabaki et al., 2021). Researchers recently analyzed 25 years of health data from nearly 8,000 middle-aged people and found a link between sleep and dementia as people get older. Middle-aged people with long-term sleep less than or equal to 6 h have a 30% increased risk of dementia (Sabia et al., 2021). However, the current research on how sleep loss promote the occurrence of dementia is still insufficient.

Sleep loss could cause cognitive dysfunction by inducing apoptosis and autophagy (Cao et al., 2019). A recent study found that the insomnia patients exhibited significant cognitive impairment and increased iron deposition in several brain regions. The iron concentration of the left hippocampus is a biomarker of cognitive impairment and may play an important role in the pathophysiological mechanism (Chen B. et al., 2019). This strongly suggests that a novel cell death mode should be considered in the process of cognitive impairment induced by sleep deprivation (SD). Ferroptosis, a recently described form of iron and lipid oxidation products-dependent regulated cell death (Dixon et al., 2012), is involved in a variety of brain disorders or injuries, including Alzheimer’s disease (Bao et al., 2021), intracerebral hemorrhage (Li et al., 2017), and traumatic brain injury (Xie et al., 2019). Dysregulation of key components of ferroptosis machinery includes iron homeostasis, inactivation of antioxidant enzymes, and lipid peroxidation (Xie et al., 2019). Insufficient sleep has been demonstrated to cause enhanced expression of genes related to oxidative stress and immune response (Möller-Levet et al., 2013). We speculated that ferroptosis may occur during SD caused memory impairment.

The sleep-wake cycle is the most overt of the circadian rhythms, which are tightly regulated in vertebrates by the hormone melatonin (Mel) (Romero et al., 2014). Melatonin has a chelating property which contributes in reducing metal-induced toxicity (Yu et al., 2019). In addition, numerous studies have demonstrated that Mel is an effective endogenous antioxidant (Galano and Reiter, 2018), which also indirectly stimulates certain antioxidant enzymes, such as superoxide dismutase (SOD) and glutathione peroxidase (GPx) (Zhao et al., 2018). Melatonin activates two high-affinity G-protein-coupled receptors—MT1 and MT2—exerting beneficial effects in circadian and sleep abnormalities (Burgess et al., 2010), mood disorders (De Crescenzo et al., 2017), learning and memory (Jilg et al., 2019), neuroprotection (Jumnongprakhon et al., 2017), and cancer (Witt-Enderby et al., 2006). Melatonin decreases neuronal firing by activating the MT1 receptor promoting sleep processes (Jin et al., 2003). Conversely, Mel promotes neurogenesis and cell proliferation *via* an MT2 receptor-dependent mechanism (Chern et al., 2012). Thus Mel modulates diverse physiological events by activating MT1 and MT2 receptors, respectively. Recently, studies have reported that Mel is an excellent ferroptosis inhibitor and its anti-ferroptosis provides a potential therapeutic target for treating traumatic brain injury (Rui et al., 2021). However, it remains unclear whether and how Mel treatment improves SD-induced hippocampal ferroptosis and cognitive impairment.

Therefore, the present mouse study aimed to explore 1) the effect of SD on memory impairment and hippocampal ferroptosis, 2) the alleviating effect of exogenous Mel supplementation on SD-induced cognitive disorders and hippocampal ferroptosis, and 3) the mechanisms by which Mel mediates SD-induced cognitive disorders and hippocampal ferroptosis.

## Materials and Methods

### Animal Treatment

A total of 112 male ICR mice (8 weeks of age; Vital River Laboratory Animal Technology Co. Ltd., Beijing, China) were placed in cages and maintained in standard environmental conditions of temperature (21 ± 1°C) and relative humidity (50 ± 10%), with a regular 14-h light/10-h dark cycle, while the light was turned on at 7:00 h (Zeitgeber time zero, or ZT0) and turned off at 21:00 h (ZT14). All mice had free access to food and water. Mice were divided into the following groups: 1) control group (CON, *n* = 24); 2) sleep deprivation group (SD, *n* = 24); SD + low Mel supplement group (SD + L-Mel, *n* = 24); SD + high Mel supplement group (SD + H-Mel, *n* = 24); Mel treatment group (Mel, *n* = 8); SD + Fer-1 supplement group (SD + Fer-1, *n* = 8).

An SD mouse model was established using a modified multi-platform water environment method (Gao et al., 2019). Following the methods of a previous study, we administered Mel supplementation at two concentrations: 20 mg/kg in the SD + L-Mel group and 40 mg/kg in the SD + H-Mel group (Gao et al., 2019). In addition, healthy mouse also received an injection of Mel (20 mg/kg). Absolute ethanol is used to dissolve Mel (M5250; Sigma, United States) and is eventually diluted with saline to 2% ethanol. According to a previous study (Li J. et al., 2020), Ferrostatin-1 (Fer-1, 1 mg/kg, SML0583; Sigma, United States), was dissolved in dimethyl sulfoxide (DMSO; Sigma, United States) and then diluted with saline to 0.5% DMSO. They were then administered by intraperitoneal injection once a day at ZT0 (7:00 am, light on) for 3 days.

A portion of the mice were subjected to spatial memory tests, while others were anesthetized using 10% chloral hydrate at ZT1 (08:00 am) on the day the experiment ended. The hippocampus of the latter were quickly harvested. One part of the hippocampus was fixed in 4% paraformaldehyde for tissue sectioning and morphological analysis, another part was frozen in at −80°C for histochemical analysis. All experiments were repeated twice. All experiments were conducted in accordance with the Guide to the Care and Use of Laboratory Animals published by the Animal Welfare Committee of the Agricultural Research Organization, China Agricultural University (Approval No. CAU201709112).

### Morris Water Maze

**FIGURE 1:**
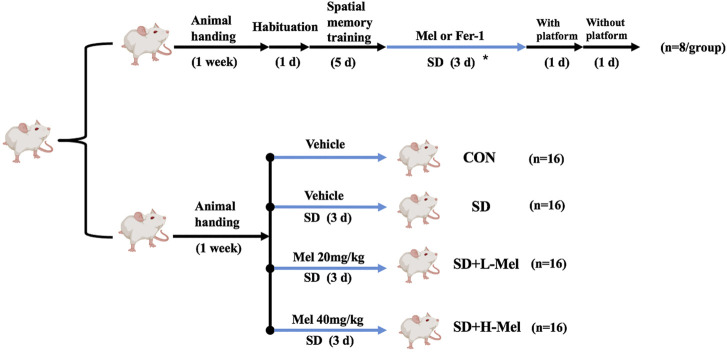
Timeline of experimental design. The mice were divided into control group (CON), sleep deprivation group (SD), SD + low melatonin (20 mg/kg) supplement group (SD + L-Mel), SD + high melatonin (40 mg/kg) supplement group (SD + H-Mel), melatonin (20 mg/kg) supplement group (Mel) and SD + Fer-1 (1 mg/kg) supplement group (SD + Fer-1). The asterisk represents mice that were subjected to 72 h acute sleep deprivation and received treatment with or without Mel or Fer-1 supplementation.

**FIGURE 2:**
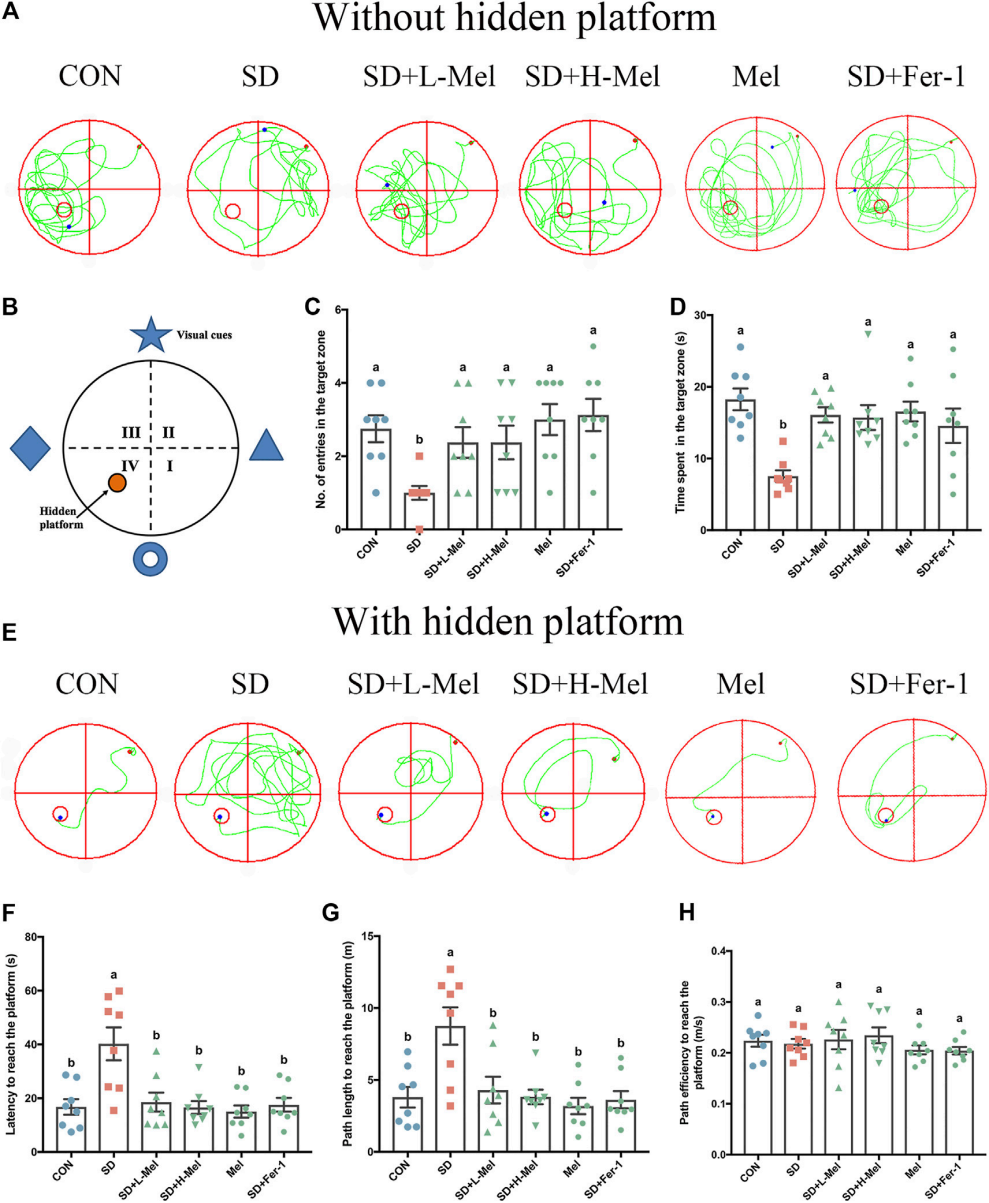
Effect of melatonin on ability of learning and memory in sleep-deprived mice. **(A)** Track plot of spatial memory test (without hidden platform). **(B)** Schematic of the Morris water maze. **(C)** Number of entries into the target zone (*n* = 8). **(D)** Time spent in the target zone (*n* = 8). **(E)** Track plot of spatial memory test (with hidden platform). **(F)** Latency to reach the platform (*n* = 8). **(G)** Path length to reach the platform (*n* = 8). **(H)** Path velocity to reach the platform (*n* = 8). Differences were assessed using one-way ANOVA. The result represents the mean ± standard error of the mean. Values not sharing a common superscript letter differ significantly at *p* < 0.05; those with the same letter do not differ significantly (*p* ≥ 0.05). CON: control group, SD: sleep deprivation group, SD + L-Mel: SD + low melatonin (20 mg/kg) supplement group, SD + H-Mel: SD + high melatonin (40 mg/kg) supplement group, Mel: melatonin (20 mg/kg) supplement group, SD + Fer-1: SD + Fer-1 (1 mg/kg) supplement group.

The Morris water maze (MWM) was used to assess spatial learning and memory abilities. The maze consisted of a round tank (120 cm in diameter, 50 cm in height) filled with warm water (23 ± 1°C) and added black nontoxic carbon ink to render opaque. The pool was divided into four quadrants (I, II, III, and IV). A moveable, hidden, circular platform was placed at a fixed location in quadrant IV and submerged about 1 cm below the water surface (Figure 2B). The timeline of the experiment is illustrated in Figure 1.

After acclimatization for a period of one week, all mice were placed in the water pool without a platform for 1 min and allowed to swim. The next all experiments were conducted at ZT1 (08:00 AM). To minimize the effects of stress on the experimental outcome, behaviorally and physically healthy mice without any stereotypical characteristics were selected for further study. On the second day, each mouse was placed in the water in all four quadrants in a fixed order to perform four training trials per day. The maximum trial duration was 60 s. Animals that failed to locate the hidden platform were manually guided. Once they reached the platform, they were allowed to remain there for 15 s. All mice received this training for five consecutive days. After 72 h of SD, mice immediately undertook the test phase (with and without the hidden platform). Learning ability was assessed on day 10. Latency (seconds), path length (m), and path velocity (mm/s) to reach the hidden platform was measured in each mouse. Memory consolidation was assessed on day 11. The platform was removed during the test, and each mouse was allowed to freely swim for 60 s. The time spent by the mice in the target zone, as well as the number of crossings over the previous platform location, were calculated. The experiment was always performed at the same time of the day under the same environmental conditions. Animal movement was tracked using a computerized tracking system (XR-XM101; Shanghai Softmaze Information Technology Co. Ltd.,).

### Cell Culture and Treatment

HT-22 cells, a sub-line derived from parent HT4 cells that were originally immortalized from primary mouse hippocampal neuronal culture. They were cultured in DMEM with 100 units/mL penicillin, 100 mg/ml streptomycin, and 10% fetal bovine serum in a CO_2_ incubator (5% CO_2_, 37°C). Cells were sub-cultured once every 3 days. Cells were plated before drug treatment in a 6- or 96-well plate at 10^6^ or 10^4^ cells/well, respectively, and cultured in DMEM with 10% FBS (complete medium) for 6 h. The cells were then cultured in DMEM without FBS (basal medium) for 12 h.

The ferroptosis inducer Erastin (E7781; Sigma, United States), the ferroptosis inhibitor Fer-1, the MT2-selective Mel receptor antagonist 4P-PDOT (1034; Tocris Bioscience, Bristol, United Kingdom), the ERK inhibitor PD98059 (1213; Tocris Bioscience, Bristol, United Kingdom) and the Nrf2 inhibitor ML385 (SML1833; Sigma, United States) were each dissolved in DMSO. Melatonin was dissolved in absolute ethyl alcohol, and further dilutions were made in basal medium to reach a final concentration. In addition, the cells were pretreated for 30 min using 10 μM 4P-PDOT, 10 μM PD98059 or 5 μM ML385, followed by 1 μM Mel or 2 μM Fer-1 for 30 min prior to Erastin (1 μM) exposure for 24 h. Cells were seeded in 96-well plates for viability assay, 6-well plates for antioxidant activity and signaling pathways assay.

Cell viability was evaluated with the 3-(4,5-dimethyl-2-thiazolyl)-2,5-diphenyl-2H-tetrazolium bromide (MTT) assay. After drug treatment, 10 μL MTT (M2003; Sigma, United States) was added to the medium (5 mg/ml in basal medium). Four hours later, the medium was discarded, and 150 μL DMSO was added to each well. The optical density of each well was measured at a wavelength of 570 nm using an enzyme-linked immune detector (550; BIO-RAD, United States). The results are expressed as a percentage of the MTT absorbance of control cells, which was set to 100%. The MTT experiment was repeated three times, with five replicates per series.

### Assessment of Antioxidant Status and Lipid Peroxidation

Hippocampal tissues or cultured HT-22 cells (*n* = 6) were homogenized in ice-cold phosphate-buffered saline (PBS) (pH 7.4). The supernatants were then extracted by centrifugation (2000 × g for 10 min) at 4°C and stored at −80°C to allow assay of superoxide dismutase (SOD) and malondialdehyde (MDA) levels using commercial kits (S0131 and S0101; Beyotime, Beijing, China). The protein concentration was determined using the BCA protein assay kit (P0012; Beyotime, Beijing, China). The MDA level and SOD activity were detected by chemical colorimetric analysis, following the manufacturer’s instructions. Values were calculated using optical density at 550 nm for SOD (expressed as units/mg of protein) and at 532 nm for MDA (expressed as μmol/mg of protein). Each sample was assayed three times.

The levels of reactive oxygen species (ROS) in the hippocampus or HT-22 cells (*n* = 6) were detected using a commercial assay kit (E004-1-1, Nanjing Jiancheng Co. Ltd., Nanjing, China). The hippocampus was washed twice using pre-cold PBS, sheared, and digested using 0.25% trypsin in a shaking water bath for 30 min at 37°C. The homogenate was then filtered through a 300-mesh stainless steel filter. Next, all cells were collected and washed twice in PBS. The cell concentration was then adjusted to 1 × 10^5^ cells/mL and the cells were loaded with 2′–7′-dichlorofluorescein diacetate (DCFH-DA) (10 μM) for 30 min at 37°C in the dark. Fluorescence intensity was detected at 502/530 nm (excitation/emission) using a fluorescence microscope reader (Synergy HT; BioTek, United States) and expressed as units of fluorescence per mg of protein. ROS generation was determined as the percentage ROS compared to control for hippocampal tissues, or as the fluorescent intensity normalized to controls for cultured HT-22 cells. Each sample was assayed three times.

For fluorescence staining of ROS, the HT-22 cells in 6-well cell culture plates were loaded with 10 μM DCFH-DA for 30 min and stained with DAPI (20 μg/ml, D8417; Sigma) for 10 min in the dark at 37°C. The cells were rinsed using PBS and fluorescence images of intracellular ROS were acquired using fluorescence microscopy (BX51; Olympus, Japan).

### Western Blot Assay

The hippocampus tissues or HT-22 cells (*n* = 6) were rapidly isolated and lysed in RIPA lysis buffer (CW2333S; CWBIO, Beijing, China) containing 1% protease inhibitor cocktail (CW2200S; CWBIO, Beijing, China) and 1% phosphatase inhibitor cocktail (CW2383S; CWBIO, Beijing, China). The lysates were centrifuged at 14,000 × g for 15 min at 4°C. The supernatants were collected, and the amount of protein was measured using a bicinchoninic acid kit (CW0014; CWBIO, Beijing, China), before the protein concentration was standardized. The protein samples were resolved using 10% sodium dodecyl sulfate-polyacrylamide gel electrophoresis (SDS-PAGE) and electro blotted onto a polyvinylidene fluoride membrane (Millipore; Billerica). Nitrocellulose membranes were blocked for 60 min using TBST [a mixture of Tris-buffered saline (TBS) and 0.05% Tween-20] containing 5% fat-free dry milk. They were then incubated in rabbit primary antibodies (MT1, 1:500, ab203038, Abcam; MT2, 1:500, ab203346, Abcam; *p*-ERK1/2, 1:500, 9109, CST; GPX4, 1:1,000, ab125066, Abcam; Nrf2, 1:1,000, ab137550, Abcam; TFR1, 1:1,000, ab214039, Abcam; DMT1, 1:1,000, NBP1-91840, Novus; FPN, 1:1,000, NBP1-21502SS, Novus; β-actin, 1:5,000, ab8227, Abcam, United States) overnight at 4°C. After washing in TBST, they were incubated in horseradish peroxidase conjugated goat anti-rabbit IgG (1:5,000; CW0103; CWBIO, Beijing, China) for 2 h at 37°C. The protein bands were detected using an enhanced chemiluminescence kit (CW0049; CWBIO, Beijing, China). The protein band intensities were quantified using ImageJ software (version 1.4, National Institutes of Health, Bethesda). The protein level was normalized to the density ratio of β-actin, while the relative protein level in the CON group *in vivo* or in the control cells *in vitro* was defined as 100%. Each sample was assayed three times.

### Real-Time Reverse Transcription-Polymerase Chain Reaction (RT-PCR)

Total RNA was isolated from the hippocampus and HT-22 cells (*n* = 6) using TRIzol reagent (CW0580A; CWBIO, Beijing, China). First strand cDNA was synthesized using the RevertAid first strand cDNA synthesis kit (K1622; Thermo Fisher, Boston, United States). RT-PCR amplification was performed using the AceQ qPCR SYBR green master mix (Q111-02; Vazyme Biotech, United States). Each sample was tested in triplicate, and relative mRNA levels were normalized to the expression levels of the housekeeping gene GAPDH. The RT-PCR primers are listed in Supplementary Table S1.

### Improved Perls’ Prussian Blue Staining

Improved iron staining based on Prussian blue staining was used in the present study (Zhang et al., 2018). Serial paraffin cross sections of brains were cut (*n* = 6; thickness: 10 μm). Sections were immersed in 0.1-M TBS (pH = 7.4) containing 3% hydrogen peroxide (H_2_O_2_) for 10 min. Next, the sections were treated using an equal ratio mixture of 4% aqueous potassium ferrocyanide and 4% hydrochloric acid for 30 min. They were rinsed using double-distilled water for 5 min, and the iron staining was amplified using TBS containing 0.025% 3′,3-diaminobenzidine tetrahydrochloride (DAB; Sigma, United States) and 0.0033% H_2_O_2_ for 10 min. This step was performed under a microscope, and the reaction was stopped when brown granules were observed in the iron deposit regions. All sections were handled in parallel to maintain the consistency of dyeing conditions. Next, the sections were stained with hematoxylin, differentiated, and sealed. Finally, the sections were observed and photographed under a microscope (BX51; Olympus, Japan). For each mouse, representative coronal brain sections with similar coordinates (Bregma—2.0 mm) were selected, five slices each from five animals/group were used in the analysis. ImageJ software (version 1.4, National Institutes of Health, Bethesda) was used to analyze the levels of iron accumulation. Results are expressed as a mean integral optical density (IOD) of iron-positive cells, normalized to that in controls.

### Immunohistochemical Staining

Paraffin sections were incubated in rabbit anti-MT2 primary antibody overnight at 4°C (1:500; ab203346; Abcam). The sections were then rinsed in 0.01-M PBS (pH 7.4) and incubated in biotinylated goat anti-rabbit IgG (1:300, sc-2020; Santa Cruz, CA, United States) for 2 h at room temperature. After washing, the tissues were incubated in streptavidin-horseradish peroxidase (1:300; Vector Laboratories, Burlingame, CA, United States) for 2 h at room temperature. Immunoreactivity was visualized by incubating the tissue sections in 0.01-M PBS containing 0.05% DAB and 0.003% H_2_O_2_ for 10 min in the dark. The sections were then stained with hematoxylin and mounted. Control slides without the primary antibody were examined in all cases. Immunoreactive cells presented with yellow-brown staining in the cytoplasm. The localization and distribution of immunoreactive positive materials in the hippocampus were observed using a microscope (BX51; Olympus, Japan) in accordance with a stereotaxic atlas of the mouse brain (Paxinos and Franklin, 2007).

### Statistical Analysis

The data were expressed as the mean ± standard error and analyzed using Graph Pad Prism version 7 (GraphPad Software, La Jolla, CA, United States). Experiments were performed at least in five independent biological and at least two independent technical replicates. Differences between groups were analyzed using one-way ANOVA followed by Tukey’s multiple comparisons tests. All *p*-values < 0.05 were considered statistically significant.

## Results

### Melatonin Alleviates Learning and Memory Deficits Induced by SD

In order to investigate whether ferroptosis is involved in SD-induced memory deficits and the alleviative effect of Mel on it, we performed behavioral analysis. In the first test (with a hidden platform) (Figure 2E), the latency was 140.1% higher in the SD group than in the CON group (*p* < 0.001), while the path length was 130% (*p* = 0.001) longer (Figures 2F,G). In the second test, without the hidden platform (Figure 2A), the number of entries into the target zone was 63.6% lower in the SD group than in the CON group (*p* = 0.003), while the time spent there was 58.7% (*p* < 0.001) lower (Figures 2C,D). The above results illustrated that SD could induce memory deficits. However, after Fer-1 supplementation in the SD mice, the latency was 56.4% (*p* = 0.001) lower than in the SD mice, the path length was 58.6% (*p* = 0.001) shorter, the number of entries into the target zone was 212.5% (*p* < 0.001) higher, and the time spent there was 93.2% (*p* = 0.032) higher, indicating that SD may induce ferroptosis, in turn leading to memory deficits.

Furthermore, after Mel supplementation in the SD mice, the latency was 54.0–58.9% (*p* = 0.001) lower than in the untreated mice, the path length was 50.9–56.3% (*p* = 0.001–0.005) shorter (Figures 2F,G), the number of entries into the target zone was 137.5% (*p* = 0.02) higher, and the time spent there was 108.3–113.2% (*p* = 0.005–0.008) higher (Figures 2C,D). Additionally, there was no significant difference between the Mel-treated groups and the CON group (*p* > 0.5). There was no significant difference in path velocity among all groups (Figure 2H). Whereas Mel alone had no effect on learning and memory ability in healthy mice. These results illustrated that Mel may ameliorate memory deficits induced by SD *via* inhibiting ferroptosis.

### Melatonin Alleviates Iron Homeostasis Dysregulation in Hippocampus Induced by SD

**FIGURE 3:**
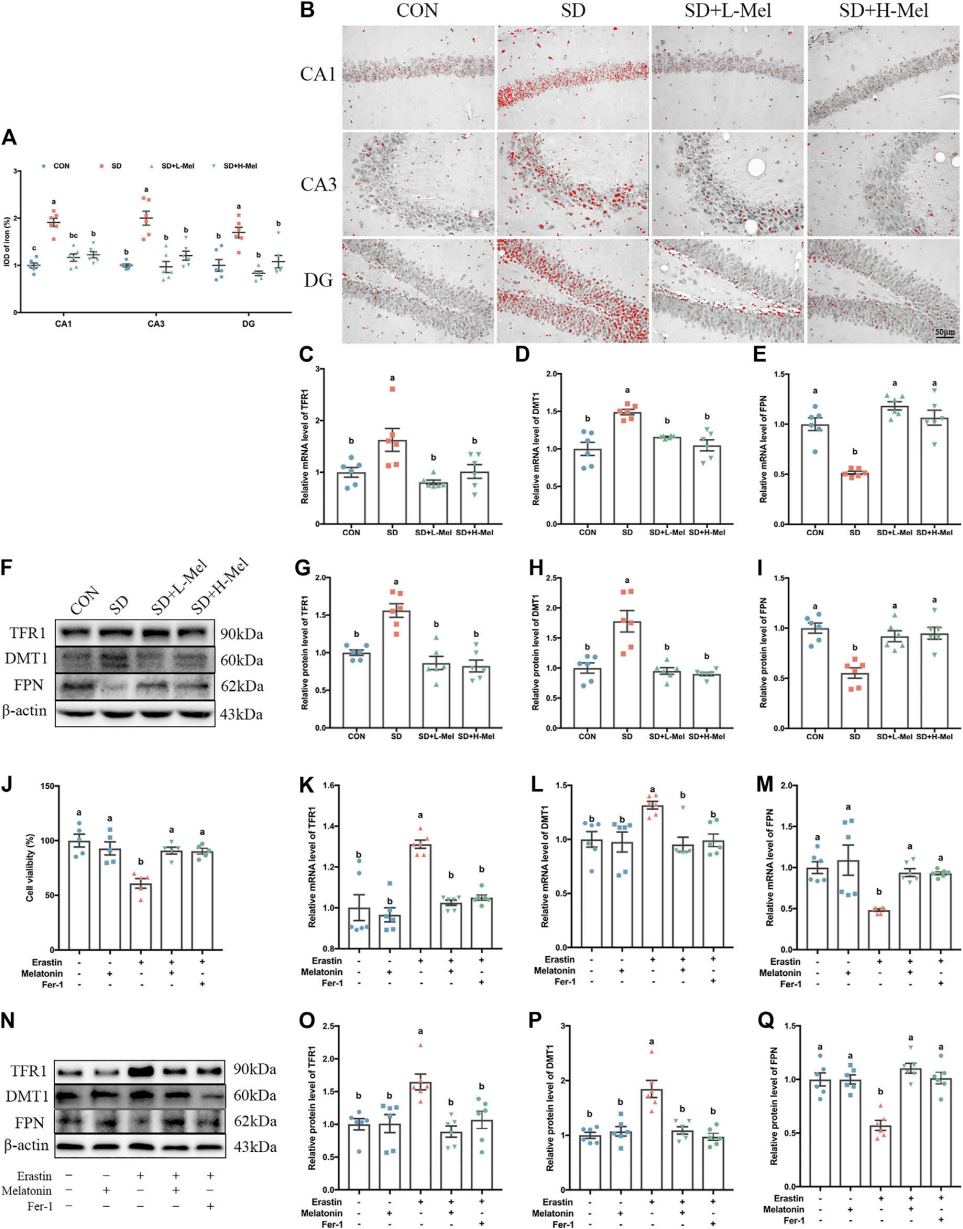
Effect of melatonin on iron accumulation and iron transporter proteins in the hippocampus of sleep-deprived mice. **(A)** Relative IOD of iron-positive cells in the hippocampal CA1, CA3, and DG areas (*n* = 6). **(B)** Micrographs depict iron labeling in mouse hippocampal sections. The iron-staining results were processed using ImageJ. Iron deposits were observed as red granules. Bar = 50 μm. **(C–E)** Relative mRNA levels of iron transporter proteins TFR1, DMT1 and FPN in the hippocampus (*n* = 6). **(F–I)** Relative protein levels of iron transporter proteins TFR1, DMT1 and FPN in the hippocampus (*n* = 6). **(J)** Relative cell viability (*n* = 5). **(K–L)** Relative mRNA levels of iron transporter proteins TFR1, DMT1 and FPN in HT-22 cells exposed to Erastin and melatonin or Fer-1 (*n* = 6). **(N–Q)** Relative protein levels of iron transporter proteins TFR1, DMT1 and FPN in HT-22 cells exposed to Erastin and melatonin or Fer-1 (*n* = 6). Differences were assessed using one-way ANOVA. The result represents the mean ± standard error of the mean. Values not sharing a common superscript letter differ significantly at *p* < 0.05; those with the same letter do not differ significantly (*p* ≥ 0.05). CON: control group, SD: sleep deprivation group, SD + L-Mel: SD + low melatonin (20 mg/kg) supplement group, SD + H-Mel: SD + high melatonin (40 mg/kg) supplement group.

One of the typical characteristics of ferroptosis is iron accumulation. To assess iron homeostasis in our model, we measured the level of iron deposition and the expression of iron homeostasis proteins. Perls staining showed that the intracellular iron ions were red and distribute the pyramidal cell of CA1-CA3 region and granule cell of DG region in the hippocampus (Figure 3B). The IOD of iron-positive cells in the hippocampal CA1, CA3, and DG areas were 91.1% (*p* < 0.001), 100.3% (*p* < 0.001), and 69.7% (*p* = 0.001) higher in the SD group than in the CON group, respectively. However, Mel supplementation attenuated the effect of SD on iron accumulation. The IOD of iron-positive cells in the hippocampal CA1, CA3, and DG areas were 39.0% (*p* < 0.001), 51.6% (*p* < 0.001), and 51.0% (*p* < 0.001) lower in the SD + L-Mel group than in the SD group, respectively. The SD + H-Mel group showed similar changes (Figure 3A).

Western blot analysis showed that the expression of transferrin receptor 1 (TFR1) in the hippocampus were 40.0% higher in the SD group than in the CON group (*p* < 0.001), while the expression of divalent metal transporter 1 (DMT1) were 77.9% higher (*p* < 0.001); conversely, ferroportin (FPN) protein levels were 44.8% lower (*p* < 0.001). Consistent with the results of the Western blot analysis, we found the mRNA levels of TFR1, DMT1 and FPN were dysregulated after SD (Figures 3C–E). However, these changes were reversed by Mel supplementation. The SD + H-Mel group showed similar changes (Figures 3G–I). These results revealed that iron deposition and the disorder of iron transporter protein in the hippocampus of SD mice were improved by Mel supplementation (Figures 3A–I).

To further verify that Mel protected against ferroptosis, we cultured HT-22 cells treated with Erastin (1 μM) with or without Mel (1 μM) or Fer-1 (2 μM) (Supplementary Figure S1). The MTT assay showed that cell viability was decreased by 39.1% (*p* < 0.001) after exposure to Erastin, but that it was increased by 49.2% (*p* = 0.001) or 48.3 (*p* = 0.002) after pretreatment with Mel or Fer-1 in Erastin-treated HT-22 cells (Figure 3J). The TFR1 protein levels were 64.7% higher in Erastin-treated HT-22 cells than in control HT-22 cells (*p* = 0.004), while DMT1 protein levels were 84.8% higher (*p* < 0.001) and FPN protein levels were 42.8% lower (*p* < 0.001). Melatonin pretreatment improved the Erastin-induced changes in TFR1, DMT1 and FPN protein and mRNA levels (Figures 3K–Q).

### Melatonin Ameliorates Lipid Peroxidation in Hippocampus Induced by SD

**FIGURE 4:**
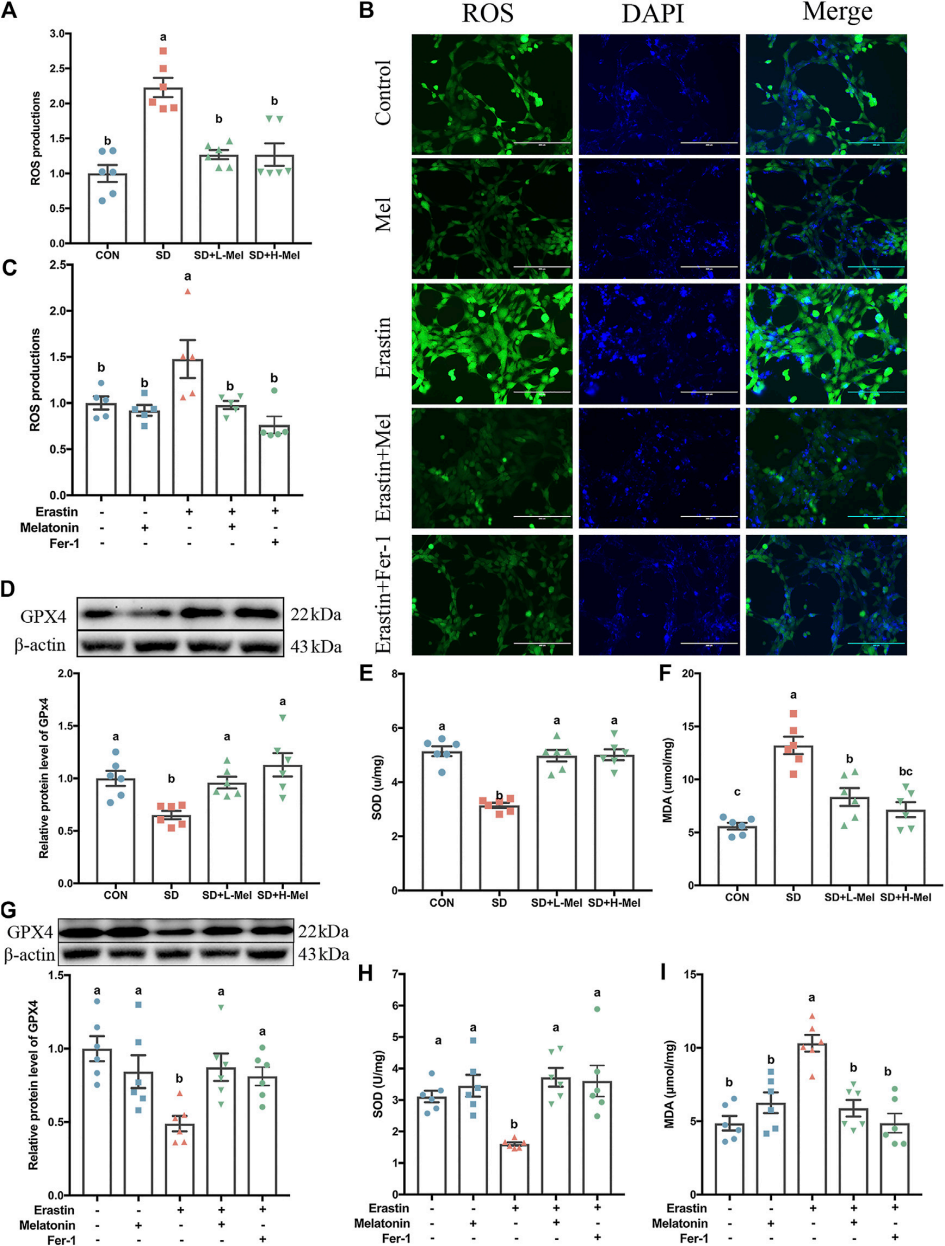
Effect of melatonin on antioxidative activation and lipid peroxidation in the hippocampus of sleep-deprived mice. **(A)** Relative quantification of the ROS assay in the hippocampus tissue (*n* = 6). **(B)** Fluorescence staining of ROS in HT-22 cells exposed to Erastin and melatonin or Fer-1. Green represents ROS, while blue represents DAPI. Bar = 200 μm. **(C)** Relative quantification of intracellular ROS level in the HT-22 cells exposed to Erastin and melatonin or Fer-1 (n = 5). **(D–F)** Relative levels of GPX4, SOD and MDA in the hippocampus (*n* = 6). **(G-I)** Relative levels of GPX4, SOD, and MDA in HT-22 cells exposed to Erastin and melatonin or Fer-1 (*n* = 6). Differences were assessed by one-way ANOVA. The result represents the mean ± standard error of the mean. Values not sharing a common superscript letter differ significantly at *p* < 0.05; those with the same letter do not differ significantly (*p* ≥ 0.05). CON: control group, SD: sleep deprivation group, SD + L-Mel: SD + low melatonin (20 mg/kg) supplement group, SD + H-Mel: SD + high melatonin (40 mg/kg) supplement group.

Another characteristic of ferroptosis is lipid peroxidation. To investigate whether SD caused lipid peroxidation in the hippocampus, we determined the levels of ROS, glutathione peroxidase 4 (GPX4), SOD, and MDA. In the SD group, the ROS levels were 122.8% higher than in the CON group (*p* < 0.001), while the MDA levels were 136.3% higher (*p* < 0.001) (Figures 4A,F). In contrast, with regards to antioxidant enzymes, GPX4 levels were 34.9% lower (*p* = 0.017), while SOD levels were 38.8% lower (*p* < 0.001) (Figures 4D,E). However, after supplementation with 20 mg/kg of Mel in SD mice, the levels of ROS were 43.1% lower than in the SD group (*p* < 0.001), while MDA levels were 36.8% lower (*p* = 0.001) (Figures 4A,F). Meanwhile, GPX4 levels were 47.5% higher (*p* = 0.038) and SOD levels were 58.1% higher (*p* < 0.001) in the SD + L-Mel group than in the SD group (Figures 4D,E). The SD + H-Mel group showed similar changes. In this regard, no significant difference occurred between the Mel-treated and CON groups (*p* > 0.053).

To further confirm the effects of Mel on Erastin-induced lipid peroxidation and antioxidative maladjustment, we determined the levels of ROS, GPX4, SOD, and MDA in the HT-22 cells. Regarding the levels of antioxidant enzymes, SOD levels were 50.5% lower in Erastin-treated HT-22 cells than in control HT-22 cells (*p* = 0.002), while GPX4 levels were 51.1% lower (*p* = 0.002) (Figures 4G,H). Consistent with these decreases in antioxidant enzymes, ROS levels were 47.8% higher in Erastin-treated HT-22 cells than in control HT-22 cells (*p* = 0.045), and MDA levels were 64.8% higher (Figures 4B,C,I) (*p* < 0.001). Melatonin pretreatment improved these Erastin-induced changes in antioxidant enzymes and lipid peroxidation (Figures 4C,G–I).

Melatonin ameliorates ferroptosis induced by SD *via* the MT2/ERK/Nrf2 signaling pathway.

**FIGURE 5:**
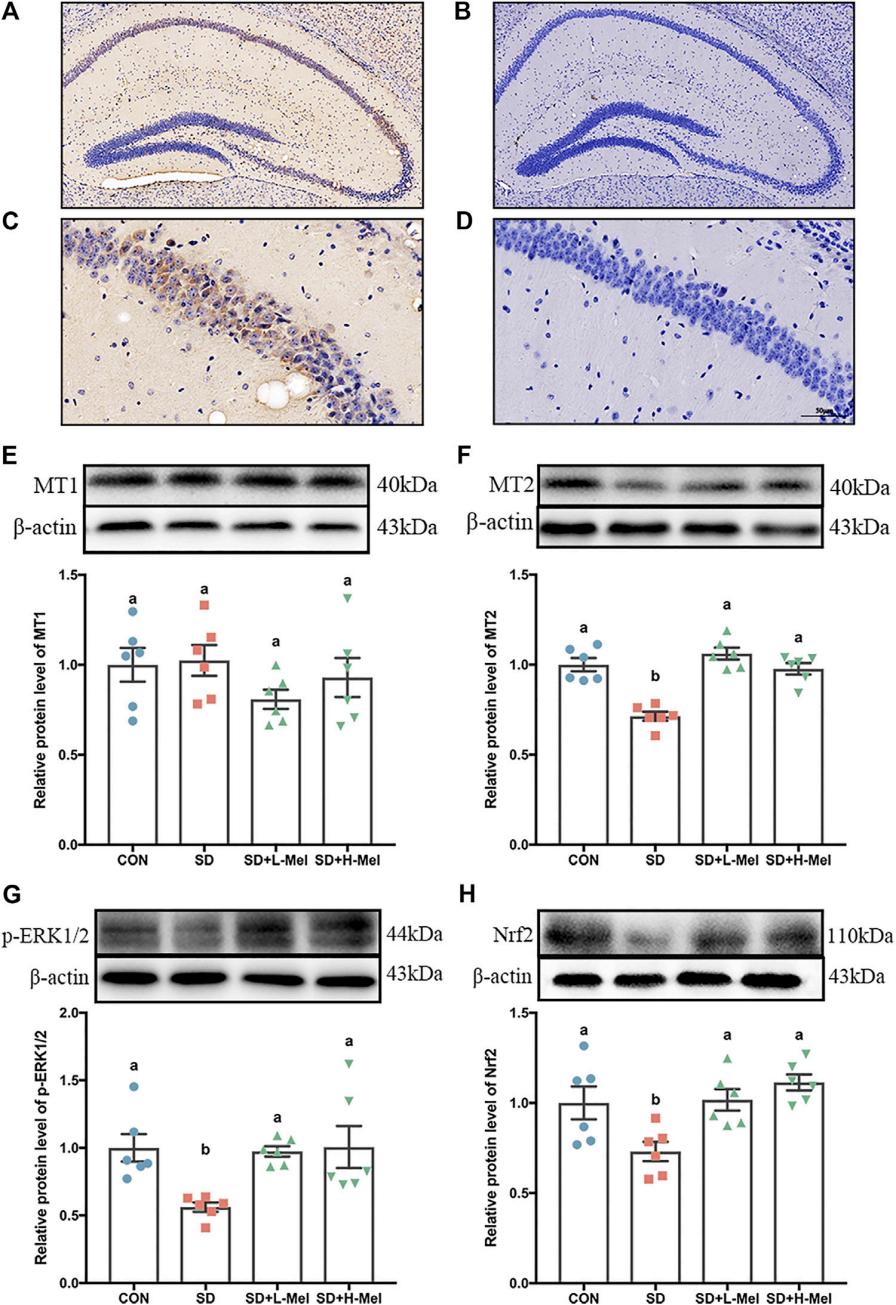
Effect of melatonin on MT2/ERK/Nrf2 signaling in the hippocampus of sleep-deprived mice **(A, C)** Immunohistochemical staining of MT2 in hippocampus. Brown indicates positive cells. **(B, D)** negative control. A–B: bar = 200 μm; C–D: bar = 50 μm. **(E–H)** Relative protein levels of MT1, MT2, *p*-ERK1/2 and Nrf2 were normalized to β-actin (*n* = 6). Differences were assessed using one-way ANOVA. The result represents the mean ± standard error of the mean. Values not sharing a common superscript letter differ significantly at *p* < 0.05; those with the same letter do not differ significantly (*p* ≥ 0.05). CON: control group, SD: sleep deprivation group, SD + L-Mel: SD + low melatonin (20 mg/kg) supplement group, SD + H-Mel: SD + high melatonin (40 mg/kg) supplement group.

**FIGURE 6:**
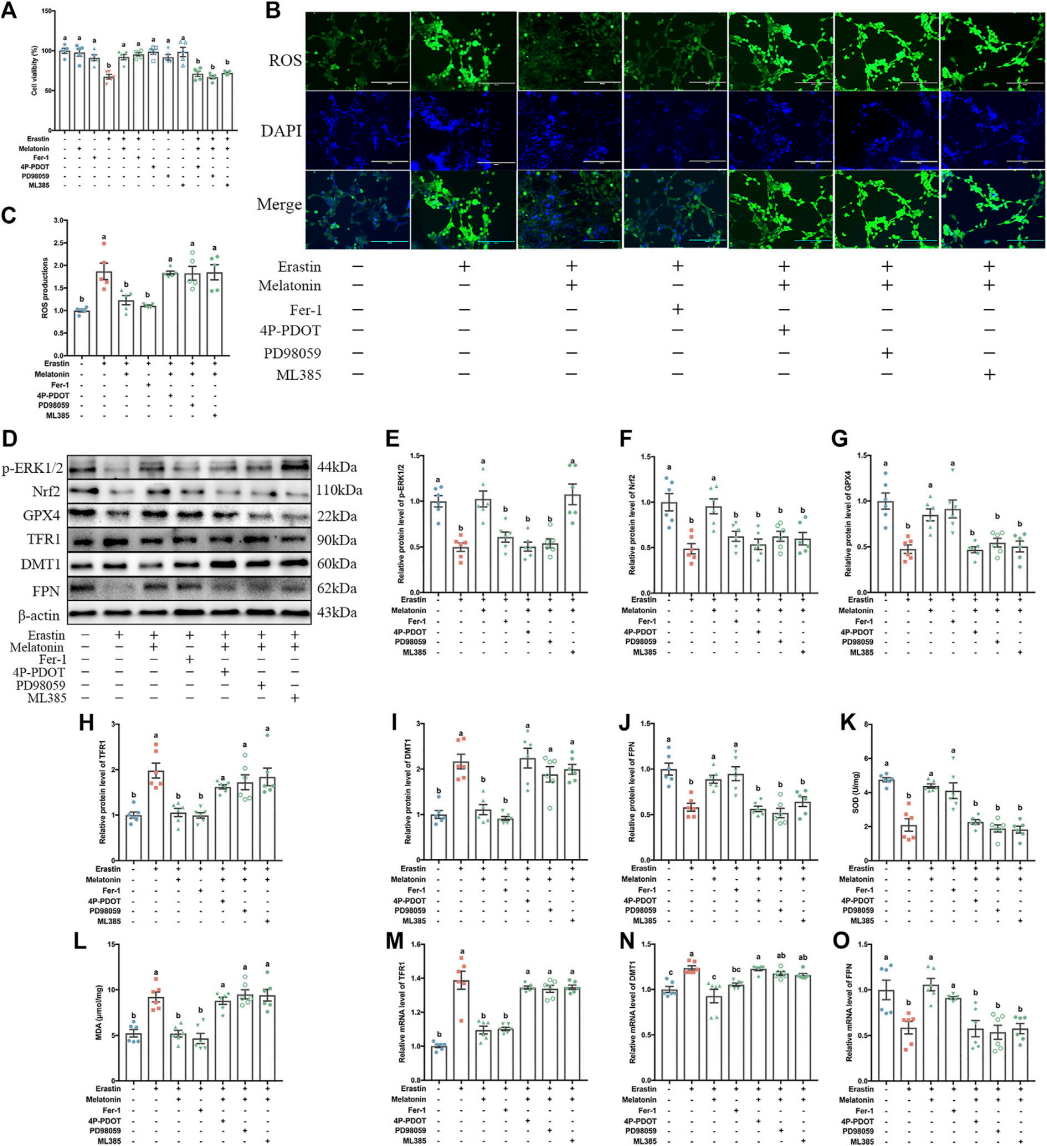
Effect of melatonin on MT2/ERK/Nrf2 signaling in the HT-22 cells exposed to Erastin. The HT-22 cells exposed to Erastin and melatonin or Fer-1 were pretreated with 4P-PDOT, PD98059 and ML385, respectively. **(A)** Relative cell viability (%) (*n* = 5). **(B)** Fluorescence staining of ROS. Bar = 200 μm. **(C)** Relative quantification of intracellular ROS level (*n* = 5). **(D–J)** Relative protein levels of *p*-ERK1/2, Nrf2, GPX4, TFR1, DMT1 and FPN normalized to β-actin (*n* = 6). **(K–L)** SOD and MDA levels (*n* = 6). **(M–O)** Relative mRNA levels of iron transporter proteins (TFR1, DMT1 and FPN) (*n* = 6). Differences were assessed using one-way ANOVA. The result represents the mean ± standard error of the mean. Values not sharing a common superscript letter differ significantly at *p* < 0.05; those with the same letter do not differ significantly (*p* ≥ 0.05).

To investigate the mechanism by which Mel alleviates SD-induced hippocampal ferroptosis, we detected the expression and action of the Mel receptors and their downstream signaling using both the *in vivo* and *in vitro* models (Figure 5, 6).

To elucidate whether the protective effect of Mel is dependent on its receptors following SD, we first determined the expression pattern of Mel receptors (MT1 and MT2) after SD. In the *in vivo*, MT2 expression was 28.6% lower in SD mice than in the CON group (*p* < 0.001). However, supplementation with 20 or 40 mg/kg Mel counteracted this SD-induced reduction in MT2 expression, increasing the levels by 36.9–48.7% (*p* < 0.001) relative to the SD group (Figure 5F). The MT2 positive cells in the hippocampal region were also assessed using immunohistochemical staining (Figures 5A–D). In contrast to MT2, MT1 expression did not differ among the treatment groups (Figure 5E).

We further examined the intracellular messenger molecules involved in MT2 action, and observed that the expression of the phosphor-extracellular signal-regulated kinase 1/2 (*p*-ERK1/2) was 43.8% lower in the SD mice than in the CON mice (*p* = 0.02), while the nuclear factor erythroid 2-related factor 2 (Nrf2) proteins were 26.9% (*p* = 0.036) lower. However, supplementation using 20 or 40 mg/kg Mel in SD mice counteracted this SD-induced reduction (Figures 5G,H). In this regard, no significant difference occurred between the Mel-treated and CON groups (*p* > 0.603).

In the *in vitro* experiment*,* to evaluate the possible involvement of Mel membrane receptors (MT2) in ferroptosis, 4P-PDOT were used in the HT-22 cells. The MTT results showed that Mel improved the Erastin-induced reduction in cell activity. This alleviating effect of Mel was blocked by pretreatment using 4P-PDOT (Figure 6A). Likewise, consistent with the changes in cell activity, *p*-ERK1/2 levels 50.2% lower (*p* < 0.001), and Nrf2 levels 51.2% lower (*p* < 0.001) in the Erastin-treated HT-22 cells than in the control HT-22 cells. In contrast, in Erastin + Mel-treated cells, *p*-ERK1/2 expression was 105.8% (*p* < 0.001) higher and Nrf2 expression was 95.6% higher (*p* = 0.001) than in Erastin-only-treated cells. This stimulating effect of Mel was blocked by pretreatment using 4P-PDOT (Figures 6E,F). To explore the role of ERK/Nrf2 signaling in the mechanism by which Mel improved Erastin-induced ferroptosis in the HT-22 cells, an ERK inhibitor (PD98059) and Nrf2 inhibitor (ML385) were used in the *in vitro* experiment. PD98059 and ML385 treatment inhibited the stimulating effect of Mel after Erastin-induced suppression of HT-22 cells activity and expression of proteins (*p*-ERK1/2 and Nrf2) (Figures 6A, D-F).

*In vitro*, we further observed the changes in antioxidant status and iron homeostasis in Erastin + Mel-treated HT-22 cells when the MT2, ERK and Nrf2 signaling pathways were inhibited. Focusing on antioxidant enzyme expression, Erastin + Mel+4P-PDOT pretreatment resulted in 44.9% lower GPX4 expression than Erastin + Mel treatment alone (*p* = 0.004), while SOD expression was 48.1% lower (*p* < 0.001) and the FPN protein levels were 36.7% lower (*p* = 0.002) (Figures 6G,J,K). With regards to lipid peroxidation products, ROS levels were 49% higher in Erastin + Mel+4P-PDOT-pretreated cells than in Erastin + Mel-treated cells (*p* = 0.019), while MDA levels were 69.2% higher (Figures 6B,C,L) (*p* < 0.001). Regarding the levels of TFR1 were 53.5% higher (*p* = 0.043), while DMT1 levels were 102.2% higher (*p* < 0.001). The results of western blot analysis and RT-PCR analysis also demonstrated the same tendency of iron transporters expression at the protein and mRNA levels (Figures 6H–J,M–O). Similar to 4P-PDOT pretreatment, PD98059 and ML385 treatment reversed the alleviating effect of Mel on the Erastin-induced changes, reducing antioxidant enzyme activation, increasing lipid peroxidation products, and causing abnormalities in iron transporter levels (Figures 6A–O).

## Discussion

Numerous studies have suggested a strong correlations between SD and cognitive impairment. But to our knowledge, this is the first study to reported that exogenous Mel treatment ameliorates hippocampal neuron ferroptosis and memory impairment induced by SD *via* the MT2/ERK/Nrf2 signaling pathway. This study provides new evidence for Mel as a drug to relieve sleep pressure.

Studies have reported that neuronal ferroptosis is responsible for AD-related memory loss (Lane et al., 2018). Using MWM test, we found that the latency and the total exercise distance necessary to reach the platform were markedly increased in SD mice, whereas the number of entries and time spent in the target zone were significantly decreased. This result suggested that SD induces memory loss in mice and corroborated previous studies in rats exposed to 48 h of SD (Wadhwa et al., 2017). Interestingly, we found that Fer-1 could improve memory impairment in sleep-deprived mice, suggesting that ferroptosis may be involved in the mechanism of SD-induced learning and memory injuries. Previous studies have reported that Fer-1, as a strong antioxidant, inhibits ferroptosis by scavenging lipid hydroperoxides in the presence of reduced iron (Miotto et al., 2020). Melatonin is also a strong antioxidant, as expected, the spatial memory disorder caused by SD was reversed by exogenous Mel supplementation. In present study, the dose of melatonin supplementation was selected based on the published literatures (Vakilzadeh et al., 2016; Zhang et al., 2017) and our preliminary screening. For example, oral administration of melatonin (30 mg/kg body weight per day for 7 days) has a protective action against BPA-induced deterioration of oocyte quality in mice (Zhang et al., 2017). Intraperitoneal injection of 50 mg/kg melatonin in C57BL/6J mice can improve the demyelination and motor behavior deficits induced by cuprizone diet (Vakilzadeh et al., 2016). These results gives us some hints that Mel may ameliorate memory impairment induced by SD *via* inhibiting ferroptosis.

To confirm our hypothesis, we further examined the effect of Mel on ferroptosis-related indicators. However, the study of ferroptosis is still in its infancy, and there is currently no easy or accurate way to detect the presence of ferroptosis. but the weakened antioxidant capacity, iron overload and the accumulation of lipid peroxidation products were the characteristic indicators of ferroptosis (Li N. et al., 2020). Perls staining showed characteristic intracellular iron accumulation in the hippocampus of SD mice in the present study. This phenomenon was reversed by Mel supplementation. The results were supported by previous studies showing that intracerebral hemorrhage can lead to an escalation in intracellular iron levels, resulting in severe neurological deficits, memory impairment, and brain atrophy (Chen L. et al., 2019). Since the brain is a lipid-enriched organ, it is highly vulnerable to iron-induced lipid peroxidation. In acute brain pathologies such as stroke, the blood-brain barrier is disrupted, and iron pools from the blood gain sudden access to the brain parenchyma. This is an important mechanism of stroke-induced neurodegeneration (DeGregorio-Rocasolano et al., 2019). Furthermore, some reports have suggested that reducing iron overload in neurons can improve hemin-induced intracerebral hemorrhage (Zhou et al., 2017). Consequently, the present data further indicated that Mel protects against SD-induced iron overload in the hippocampus.

Intracellular iron levels are regulated by multiple iron transporter proteins. Fe^3+^ is imported into cells by the membrane protein TFR1. It then migrates to the endosome. Similarly, DMT1 mediates the release of Fe^2+^ from the endosome into a labile iron pool in the cytoplasm. Iron export is mediated by the membrane protein FPN, ceruloplasmin can oxidize Fe^2+^ to Fe^3+^ (Hao et al., 2018). Each brain cell type maintains their internal iron homeostasis by changing their expression of proteins involved in iron uptake, efflux, storage, and mobilization. Therefore, correcting imbalances in iron transporters is key to maintaining intracellular iron homeostasis (Masaldan et al., 2019). In the present study, Mel supplementation significantly improved the SD-induced up-regulation of TFR1 and DMT1, as well as the down-regulation of FPN. As a result, intracellular iron accumulation in the hippocampus was suppressed. These results corroborated a study by Zhang et al., which found that a disorder of various iron transporters resulted in ferroptosis in the hippocampus and cortex of P301S mice—an Alzheimer’s model (Zhang et al., 2018). Importantly, in the *in vitro* experiment, we found that Mel or Fer-1 reversed Erastin-induced iron transporter abnormalities in HT-22 cells. Consequently, both our *in vivo* and *in vitro* experiments revealed that Mel alleviates SD-induced hippocampal ferroptosis by improving iron transporter abnormalities.

Excess active iron donates electrons, leading to ROS generation through the Fenton reaction, promoting lipid peroxidation and initiating ferroptosis (Dixon and Stockwell, 2014). Many researchers believe that ROS and MDA accumulation reflect the progress of cellular ferroptosis (Yamada et al., 2020; Yuan et al., 2020), while GPX4 and SOD are key antioxidant enzymes for scavenging excess ROS and MDA (Galaris et al., 2019). The role of GPX4 in ferroptosis is particularly important, studies reported that deletion of GPX4 in forebrain neurons promotes cognitive impairment and neurodegeneration (Hambright et al., 2017). In the present study, consistent with the accumulation of iron, we observed an increase in ROS and MDA levels, as well as a reduction in SOD and GPX4 expression in the hippocampus of SD mice. These effects were reversed by Mel supplementation. Similar to the *in vivo* results, Mel or Fer-1 reversed the Erastin-induced decreases in GPX4 and SOD and increases in ROS and MDA in HT-22 cells *in vitro*, suggesting that Mel can increase the activity of antioxidant enzymes (GPX4 and SOD) to suppress oxidative stress and ferroptosis in the hippocampus of SD mice. These results confirmed our previous speculation, Mel could ameliorate memory impairment induced by SD *via* inhibiting ferroptosis.

Next, we further explored the mechanism by which Mel exited its ameliorative effect. Melatonin modulates numerous physiological processes in mammals through its two receptors: MT1 and MT2 (Pandi-Perumal et al., 2008). However, in our *in vivo* experiment, SD reduced MT2 expression in the mouse hippocampus; this inhibitory effect of SD was reversed by Mel supplementation, but no change in MT1 expression occurred in SD mice, regardless of whether Mel supplementation was administered. The *in vitro* experiment further confirmed that Mel pretreatment inhibited the ferroptosis caused by Erastin in HT-22 cells, and that the inhibitory effect of Mel was blocked by the MT2-specific antagonist 4P-PDOT. Additionally, some researchers have carried out a more in-depth discussion. They combined the addition of melatonin with 4P-PDOT in a model of cerebral ischemia, the results showed that the addition of 4P-PDOT counterbalanced the neuroprotective effect of melatonin on ferroptosis induced by cerebral ischemia (Rui et al., 2021). However, melatonin not only binds to the membrane receptors, but also directly pass through the cell membrane and bind to the nuclear receptors of the retinoid-related orphan nuclear hormone receptor (RORα/RZR) family to regulate transcription factors in the nucleus (Zhao et al., 2019). For mammals, quinone reductase also acts as a binding site for melatonin and plays an important role in balancing generation of free radicals (Gurunathan et al., 2020). In general, our study demonstrates that the MT2 receptor is the major melatonin receptor subtype involved in melatonin’s protection against SD-induced ferroptosis. However, whether other mechanisms are involved in the protective effect of melatonin on ferroptosis caused by SD need to be further investigation.

The MT2 receptor exerts its physiological effects through downstream signaling pathways such as MAPKs/ERK and Nrf2 (Pandi-Perumal et al., 2008). In the present study, the *in vivo* experiment showed a reduction in *p*-ERK, and Nrf2 expressions in the SD mice, consistent with the decreases in hippocampal MT2 levels. However, these decreases were reversed by Mel supplementation. Likewise, the *in vitro* experiment showed that Mel had a stimulatory effect by attenuating the Erastin-induced down-regulation of *p*-ERK, and Nrf2 expression. This effect of Mel was blocked by the MT2-specific antagonist 4P-PDOT in HT-22 cells. Several reports have suggested that Erastin-induced ferroptosis is alleviated through the ERK pathway in mouse hippocampal HT-22 cells (Hirata et al., 2019). Furthermore, Nrf2 plays a critical role in ferroptosis (Dodson et al., 2019). Principal proteins and enzymes engaged in ferroptosis induction and inhibition are encoded by Nrf2 target genes. Interestingly, in our *in vitro* experiment, PD98059 and ML385 blocked the rescuing effect of Mel on the Erastin-induced reduction in antioxidant enzymes. Furthermore, these inhibitors led to an increase in lipid peroxidation and abnormalities in iron transporters. In contrast, 4P-PDOT and PD98059 treatment blocked the up-regulatory effect of Mel on *p*-ERK, whereas ML385 induced no changes. A previous study reported that gastrodin can ameliorate oxidative stress and ferroptosis caused by glutamate in HT-22 cells through the Nrf2/HO-1 signaling pathway (Jiang et al., 2020), and several reports have stated that Mel attenuates the oxidative stress and memory impairment associated with klotho deficiency through a signaling interaction between the MT2 receptor and Nrf2-related antioxidant effects (Shin et al., 2015). In this context, our findings suggested that Mel activates the ERK signaling pathway through the MT2 receptor pathway, which in turn activates the expression of Nrf2 and downstream signaling molecules, inhibiting SD-induced ferroptosis.

**FIGURE 7:**
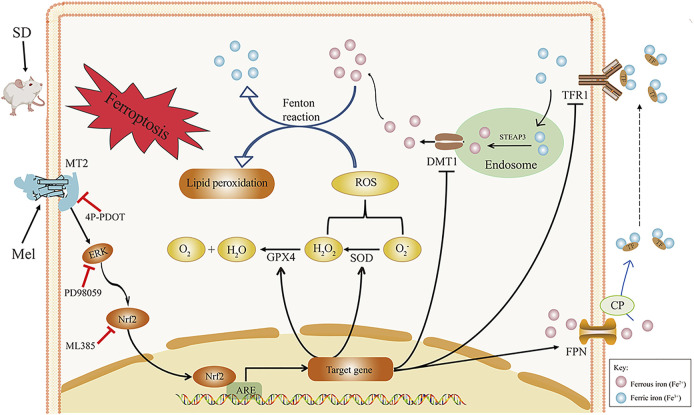
Hypothetical diagram of how melatonin improves SD-induced hippocampal ferroptosis. Exogenous melatonin likely alleviates hippocampal ferroptosis caused by acute SD through by binding to the MT2 receptor and activating ERK/Nrf2 signaling, thereby improving lipid peroxidation and iron transporter disorder. ARE, antioxidant response element; CP, ceruloplasmin; DMT1, divalent metal transporter 1; ERK1/2, extracellular regulated protein kinases; FPN, ferroportin; GPX4, glutathione peroxidase 4; Keap1, Kelch-like ECH-associated protein 1; Mel, melatonin; ML385, Nrf2 inhibitor; MDA, malondialdehyde; MT2, melatonin receptor 2; Nrf2, nuclear factor erythroid 2-related factor 2; PD98059, ERK inhibitor; ROS, reactive oxygen species; SD, sleep deprivation; SOD, superoxide dismutase; STEAP3, 6-transmembrane epithelial antigen of the prostate 3; TFR1, transferrin receptor 1; 4P-PDOT, 4-phenyl-2-propionamidotetralin.

In summary, acute SD led to an increase in intracellular iron accumulation and lipid peroxidation in the hippocampus, which resulted in hippocampal ferroptosis and memory impairment. However, Mel supplementation in SD mice reversed these changes. Administering exogenous Mel alleviated acute SD-induced memory loss, perhaps because Mel binds to the MT2 receptor in the hippocampus, activating ERK phosphorylation, as well as the Nrf2 signaling pathway, to down-regulate TFR1 and DMT1, up-regulate FPN, and activate GPX4 and SOD. This should improve iron transporter abnormalities and lipid peroxidation to suppress hippocampal ferroptosis, as summarized in Figure 7. Our finds suggest that Mel could be used as an effective drug to protect against SD-induced memory loss.

## Data Availability

The original contributions presented in the study are included in the article/Supplementary Material, further inquiries can be directed to the corresponding author.

## References

[B1] Bao W.-D. Pang P. Zhou X.-T. Hu F. Xiong W. Chen K. (2021). Loss of ferroportin induces memory impairment by promoting ferroptosis in Alzheimer's disease. Cell Death Differ 28 (5), 1548–1562. 10.1038/s41418-020-00685-9 33398092 PMC8166828

[B2] Burgess H. J. Revell V. L. Molina T. A. Eastman C. I. (2010). Human Phase Response Curves to Three Days of Daily Melatonin: 0.5 mgVersus3.0 mg. J. Clin. Endocrinol. Metab. 95 (7), 3325–3331. 10.1210/jc.2009-2590 20410229 PMC2928909

[B3] Cao Y. Li Q. Liu L. Wu H. Huang F. Wang C. (2019). Modafinil protects hippocampal neurons by suppressing excessive autophagy and apoptosis in mice with sleep deprivation. Br. J. Pharmacol. 176 (9), 1282–1297. 10.1111/bph.14626 30767208 PMC6468261

[B4] Chen B. Chen Z. Liu M. Gao X. Cheng Y. Wei Y. (2019a). Inhibition of neuronal ferroptosis in the acute phase of intracerebral hemorrhage shows long-term cerebroprotective effects. Brain Res. Bull. 153, 122–132. 10.1016/j.brainresbull.2019.08.013 31442590

[B5] Chen L. Wei X. Liu C. Li C. Zhou Z. (2019b). Brain iron deposition in primary insomnia-An *in vivo* susceptibility‐weighted imaging study. Brain Behav. 9 (1), e01138. 10.1002/brb3.1138 30548431 PMC6346654

[B6] Chern C.-M. Liao J.-F. Wang Y.-H. Shen Y.-C. (2012). Melatonin ameliorates neural function by promoting endogenous neurogenesis through the MT2 melatonin receptor in ischemic-stroke mice. Free Radic. Biol. Med. 52 (9), 1634–1647. 10.1016/j.freeradbiomed.2012.01.030 22330064

[B7] De Crescenzo F. Lennox A. Gibson J. C. Cordey J. H. Stockton S. Cowen P. J. (2017). Melatonin as a treatment for mood disorders: a systematic review. Acta Psychiatr. Scand. 136 (6), 549–558. 10.1111/acps.12755 28612993

[B8] DeGregorio-Rocasolano N. Martí-Sistac O. Gasull T. (2019). Deciphering the iron side of stroke: neurodegeneration at the crossroads between iron dyshomeostasis, excitotoxicity, and ferroptosis. Front. Neurosci. 13, 85. 10.3389/fnins.2019.00085 30837827 PMC6389709

[B9] Dixon S. J. Lemberg K. M. Lamprecht M. R. Skouta R. Zaitsev E. M. Gleason C. E. (2012). Ferroptosis: an iron-dependent form of nonapoptotic cell death. Cell 149 (5), 1060–1072. 10.1016/j.cell.2012.03.042 22632970 PMC3367386

[B10] Dixon S. J. Stockwell B. R. (2014). The role of iron and reactive oxygen species in cell death. Nat. Chem. Biol. 10 (1), 9–17. 10.1038/nchembio.1416 24346035

[B11] Dodson M. Castro-Portuguez R. Zhang D. D. (2019). NRF2 plays a critical role in mitigating lipid peroxidation and ferroptosis. Redox Biol. 23, 101107. 10.1016/j.redox.2019.101107 30692038 PMC6859567

[B12] Galano A. Reiter R. J. (2018). Melatonin and its metabolites vs oxidative stress: from individual actions to collective protection. J. Pineal Res. 65 (1), e12514. e12514. 10.1111/jpi.12514 29888508

[B13] Galaris D. Barbouti A. Pantopoulos K. (2019). Iron homeostasis and oxidative stress: an intimate relationship. Biochim. Biophys. Acta (Bba) - Mol. Cel Res. 1866 (12), 118535. 10.1016/j.bbamcr.2019.118535 31446062

[B14] Gao T. Wang Z. Dong Y. Cao J. Lin R. Wang X. (2019). Role of melatonin in sleep deprivation‐induced intestinal barrier dysfunction in mice. J. Pineal Res. 67 (1), e12574. e12574. 10.1111/jpi.12574 30929267

[B15] Gurunathan S. Kang M.-H. Kim J.-H. (2020). Role and therapeutic potential of melatonin in the central nervous system and cancers. Cancers 12 (6), 1567. 10.3390/cancers12061567 PMC7352348 32545820

[B16] Hambright W. S. Fonseca R. S. Chen L. Na R. Ran Q. (2017). Ablation of ferroptosis regulator glutathione peroxidase 4 in forebrain neurons promotes cognitive impairment and neurodegeneration. Redox Biol. 12, 8–17. 10.1016/j.redox.2017.01.021 28212525 PMC5312549

[B17] Hao S. Liang B. Huang Q. Dong S. Wu Z. He W. (2018). Metabolic networks in ferroptosis (Review). Oncol. Lett. 15 (4), 5405–5411. 10.3892/ol.2018.8066 29556292 PMC5844144

[B18] Hirata Y. Iwasaki T. Makimura Y. Okajima S. Oh-hashi K. Takemori H. (2019). Inhibition of double-stranded RNA-dependent protein kinase prevents oxytosis and ferroptosis in mouse hippocampal HT22 cells. Toxicology 418, 1–10. 10.1016/j.tox.2019.02.012 30817950

[B19] Jiang T. Cheng H. Su J. Wang X. Wang Q. Chu J. (2020). Gastrodin protects against glutamate-induced ferroptosis in HT-22 cells through Nrf2/HO-1 signaling pathway. Toxicol. Vitro 62, 104715. 10.1016/j.tiv.2019.104715 31698019

[B20] Jilg A. Bechstein P. Saade A. Dick M. Li T. X. Tosini G. (2019). Melatonin modulates daytime-dependent synaptic plasticity and learning efficiency. J. Pineal Res. 66 (3), e12553, 2019. e12553. 10.1111/jpi.12553 PMC6405292 30618149

[B21] Jin X. von Gall C. Pieschl R. L. Gribkoff V. K. Stehle J. H. Reppert S. M. (2003). Targeted Disruption of the Mouse Mel 1b Melatonin Receptor. Mol. Cel. Biol. 23 (3), 1054–1060. 10.1128/mcb.23.3.1054-1060.2003 PMC140714 12529409

[B22] Jumnongprakhon P. Sivasinprasasn S. Govitrapong P. Tocharus C. Tocharus J. (2017). Activation of melatonin receptor (MT1/2) promotes P-gp transporter in methamphetamine-induced toxicity on primary rat brain microvascular endothelial cells. Toxicol. Vitro 41, 42–48. 10.1016/j.tiv.2017.02.010 28223141

[B23] Lane D. J. R. Ayton S. Bush A. I. (2018). Iron and Alzheimer's disease: an update on emerging mechanisms. Jad 64 (s1), S379–s395. 10.3233/jad-179944 29865061

[B24] Leng Y. McEvoy C. T. Allen I. E. Yaffe K. (2017). Association of Sleep-Disordered Breathing With Cognitive Function and Risk of Cognitive Impairment. JAMA. Neurol. 74 (10), 1237–1245. 10.1001/jamaneurol.2017.2180 28846764 PMC5710301

[B25] Li J. Cao F. Yin H.-l. Huang Z.-j. Lin Z.-t. Mao N. (2020a). Ferroptosis: past, present and future. Cell Death Dis. 11 (2), 88. 10.1038/s41419-020-2298-2 32015325 PMC6997353

[B26] Li N. Wang W. Zhou H. Wu Q. Duan M. Liu C. (2020b). Ferritinophagy-mediated ferroptosis is involved in sepsis-induced cardiac injury. Free Radic. Biol. Med. 160, 303–318. 10.1016/j.freeradbiomed.2020.08.009 32846217

[B27] Li Q. Han X. Lan X. Gao Y. Wan J. Durham F. (2017). Inhibition of neuronal ferroptosis protects hemorrhagic brain. JCI. Insight 2 (7), e90777. 10.1172/jci.insight.90777 28405617 PMC5374066

[B28] Masaldan S. Bush A. I. Devos D. Rolland A. S. Moreau C. (2019). Striking while the iron is hot: iron metabolism and ferroptosis in neurodegeneration. Free Radic. Biol. Med. 133, 221–233. 10.1016/j.freeradbiomed.2018.09.033 30266679

[B29] Miotto G. Rossetto M. Di Paolo M. L. Orian L. Venerando R. Roveri A. (2020). Insight into the mechanism of ferroptosis inhibition by ferrostatin-1. Redox Biol. 28, 101328. 10.1016/j.redox.2019.101328 31574461 PMC6812032

[B30] Möller-Levet C. S. Archer S. N. Bucca G. Laing E. E. Slak A. Kabiljo R. (2013). Effects of insufficient sleep on circadian rhythmicity and expression amplitude of the human blood transcriptome. Proc. Natl. Acad. Sci. 110 (12), E1132–E1141. 10.1073/pnas.1217154110 23440187 PMC3607048

[B31] Pandiperumal S. Trakht I. Srinivasan V. Spence D. Maestroni G. Zisapel N. (2008). Physiological effects of melatonin: Role of melatonin receptors and signal transduction pathways. Prog. Neurobiol. 85 (3), 335–353. 10.1016/j.pneurobio.2008.04.001 18571301

[B32] Paxinos G. Franklin K. B. J. (2007). The Mouse brain in stereotaxic coordinates, 2001. San Diego: A Harcourt Science and Technology Company, 350.

[B33] Romero A. Ramos E. de Los Ríos C. Egea J. del Pino J. Reiter R. J. (2014). A review of metal-catalyzed molecular damage: protection by melatonin. J. Pineal Res. 56 (4), 343–370. 10.1111/jpi.12132 24628077

[B34] Rui T. Wang H. Li Q. Cheng Y. Gao Y. Fang X. (2021). Deletion of ferritin H in neurons counteracts the protective effect of melatonin against traumatic brain injury‐induced ferroptosis. J. Pineal Res. 70 (2), e12704. 10.1111/jpi.12704 33206394

[B35] Sabia S. Fayosse A. Dumurgier J. van Hees V. T. Paquet C. Sommerlad A. (2021). Association of sleep duration in middle and old age with incidence of dementia. Nat. Commun. 12 (1), 2289. 10.1038/s41467-021-22354-2 33879784 PMC8058039

[B36] Shahrbabaki S. S. Linz D. Hartmann S. Redline S. Baumert M. (2021). Sleep arousal burden is associated with long-term all-cause and cardiovascular mortality in 8001 community-dwelling older men and women. Eur. Heart J. 42 (21), 2088–2099. 10.1093/eurheartj/ehab151 33876221 PMC8197565

[B37] Shin E.-J. Chung Y. H. Le H.-L. T. Jeong J. H. Dang D.-K. Nam Y. (2015). Melatonin attenuates memory impairment induced by klotho gene deficiency via interactive signaling between MT2 receptor, ERK, and Nrf2-related antioxidant potential. Int. J. Neuropsychopharmacol. 18 (6), 14. 10.1093/ijnp/pyu105 PMC4438546 25550330

[B38] Vakilzadeh G. Khodagholi F. Ghadiri T. Ghaemi A. Noorbakhsh F. Sharifzadeh M. (2016). The effect of melatonin on behavioral, molecular, and histopathological changes in cuprizone model of demyelination. Mol. Neurobiol. 53 (7), 4675–4684. 10.1007/s12035-015-9404-y 26310973

[B39] Wadhwa M. Prabhakar A. Ray K. Roy K. Kumari P. Jha P. K. (2017). Inhibiting the microglia activation improves the spatial memory and adult neurogenesis in rat hippocampus during 48 h of sleep deprivation. J. Neuroinflammation 14 (1), 222. 10.1186/s12974-017-0998-z 29141671 PMC5688670

[B40] Witt-Enderby P. A. Radio N. M. Doctor J. S. Davis V. L. (2006). Therapeutic treatments potentially mediated by melatonin receptors: potential clinical uses in the prevention of osteoporosis, cancer and as an adjuvant therapy. J. Pineal Res. 41 (4), 297–305. 10.1111/j.1600-079X.2006.00369.x 17014686

[B41] Xie B. S. Wang Y. Q. Lin Y. Mao Q. Feng J. F. Gao G. Y. (2019). Inhibition of ferroptosis attenuates tissue damage and improves long‐term outcomes after traumatic brain injury in mice. CNS Neurosci. Ther. 25 (4), 465–475. 10.1111/cns.13069 30264934 PMC6488926

[B42] Yamada N. Karasawa T. Kimura H. Watanabe S. Komada T. Kamata R. (2020). Ferroptosis driven by radical oxidation of n-6 polyunsaturated fatty acids mediates acetaminophen-induced acute liver failure. Cel Death Dis 11 (2), 144. 10.1038/s41419-020-2334-2 PMC7039960 32094346

[B43] Yu H. Zhang J. Ji Q. Yu K. Wang P. Song M. (2019). Melatonin alleviates aluminium chloride-induced immunotoxicity by inhibiting oxidative stress and apoptosis associated with the activation of Nrf2 signaling pathway. Ecotoxicology Environ. Saf. 173, 131–141. 10.1016/j.ecoenv.2019.01.095 30771656

[B44] Yuan L.-Q. Wang C. Lu D.-F. Zhao X.-D. Tan L.-H. Chen X. (2020). Induction of apoptosis and ferroptosis by a tumor suppressing magnetic field through ROS-mediated DNA damage. Aging 12 (4), 3662–3681. 10.18632/aging.102836 32074079 PMC7066880

[B45] Zhang M. Dai X. Lu Y. Miao Y. Zhou C. Cui Z. (2017). Melatonin protects oocyte quality from Bisphenol A-induced deterioration in the mouse. J. Pineal Res. 62 (3), e12396. 10.1111/jpi.12396 28178360

[B46] Zhang Y.-H. Wang D.-W. Xu S.-F. Zhang S. Fan Y.-G. Yang Y.-Y. (2018). α-Lipoic acid improves abnormal behavior by mitigation of oxidative stress, inflammation, ferroptosis, and tauopathy in P301S Tau transgenic mice. Redox Biol. 14, 535–548. 10.1016/j.redox.2017.11.001 29126071 PMC5684493

[B47] Zhao C.-N. Wang P. Mao Y.-M. Dan Y.-L. Wu Q. Li X.-M. (2019). Potential role of melatonin in autoimmune diseases. Cytokine & Growth Factor. Rev. 48, 1–10. 10.1016/j.cytogfr.2019.07.002 31345729

[B48] Zhao Z. Lu C. Li T. Wang W. Ye W. Zeng R. (2018). The protective effect of melatonin on brain ischemia and reperfusion in rats and humans: *In vivo* assessment and a randomized controlled trial. J. Pineal Res. 65 (4), e12521. e12521. 10.1111/jpi.12521 30098076

[B49] Zhou Y.-F. Zhang C. Yang G. Qian Z.-M. Zhang M.-W. Ma J. (2017). Hepcidin protects neuron from hemin-mediated injury by reducing iron. Front. Physiol. 8, 332. 10.3389/fphys.2017.00332 28588503 PMC5440571

