# Supplementary material for: Melatonin Alleviates Acute Sleep Deprivation-Induced Memory Loss in Mice by Suppressing Hippocampal Ferroptosis
Source: Front Pharmacol. 2021 Jul 16;12:708645. doi: 10.3389/fphar.2021.708645 (PMC8322577; doi:10.3389/fphar.2021.708645)
Supplement: Supplementary file 1 [file DataSheet1.docx]

**Supplementary Material**

**Supplemental Table**

**Table 1 Primers for Real-time PCR**

| Gene name | Primer sequence | Produce size | Accession |
| --- | --- | --- | --- |
| TFR1 | F:TTGGGTAGTTGGAGATTGCC | 247 | NM_011638.4 |
|  | R:TGAGGTCTTTGGCTTCTGGT |  |  |
| DMT1 | F:CTGATCGTCTGCTCCATCAA | 149 | NM_001146161.1 |
|  | R:CCCAATGCAATCAAACACTG |  |  |
| FPN | F:CCCTTCCGCACTTTCCGAAT | 204 | NM_008732.2 |
|  | R:GAATAGACCAGTCCGAACAAGGC |  |  |
| GAPDH | F:CCGAGAATGGGAAGCTTGTC | 232 | NM_001289726.1 |
|  | R:TTCTCGTGGTTCACACCCATC |  |  |

**Supplemental Figure 1**


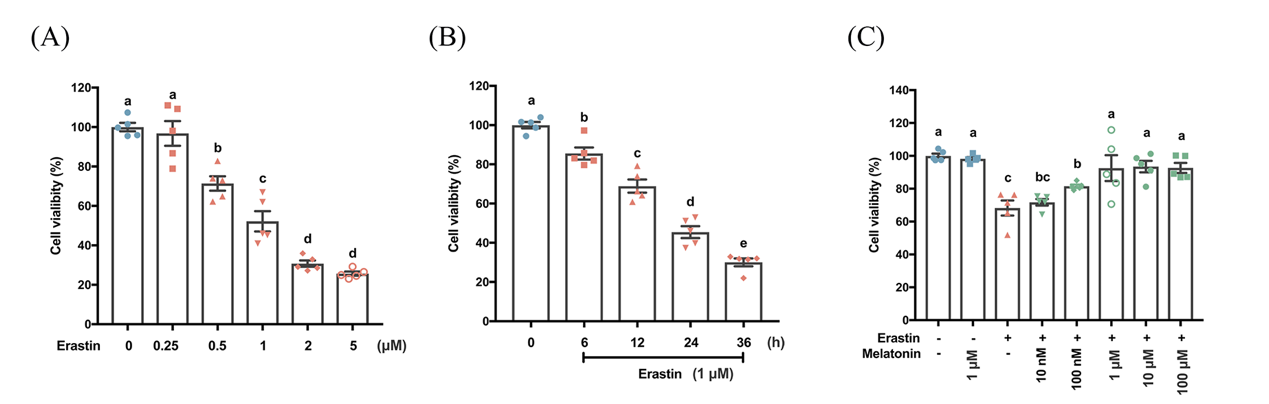


**Supplemental figure 1. Effect of melatonin on Erastin-induced cell death in HT-22 cells.** (**A**) MTT assay for the viability of HT-22 cells after treatment with Erastin (0.25, 0.5, 1, 2, 5 μM) for 24 hours. (**B**) MTT assay for the viability of HT-22 cells after treatment with 1 μM Erastin for 6, 12, 24, 36 hours. (**C**) HT-22 cells were treated with melatonin at various concentrations (10nM, 100nM, 1 μM, 10μM, 100μM) for 30 min prior to Erastin (1 μM) exposure for 24 h. Differences were assessed using one-way ANOVA. The result represents the mean ± standard error of the mean. Values not sharing a common superscript letter differ significantly at p < 0.05; those with the same letter do not differ significantly (p ≥ 0.05).
